# Comparison of Rumen Fluid pH by Continuous Telemetry System and Bench pH Meter in Sheep with Different Ranges of Ruminal pH

**DOI:** 10.1155/2014/195782

**Published:** 2014-05-21

**Authors:** Leonardo F. Reis, Antonio H. H. Minervino, Carolina A. S. C. Araújo, Rejane S. Sousa, Francisco L. C. Oliveira, Frederico A. M. L. Rodrigues, Enoch B. S. Meira-Júnior, Raimundo A. Barrêto-Júnior, Clara S. Mori, Enrico L. Ortolani

**Affiliations:** ^1^Department of Clinical Science, Faculty of Veterinary Medicine, University of Sao Paulo, Avenida Professor Orlando Marques de Paiva, 87 Cidade Universitária, 05508-270 São Paulo, SP, Brazil; ^2^Institute of Biodiversity and Forest, Federal University of Western Pará, Avenida Vera Paz S/N, Salé, 68000-000 Santarém, PA, Brazil; ^3^Department of Animal Science, Federal Rural University of the Semiarid Region, Avenida Francisco Mota, s/n Bairro Presidente Costa e Silva, 59625-900 Mossoró, RN, Brazil

## Abstract

We aimed to compare the measurements of sheep ruminal pH using a continuous telemetry system or a bench pH meter using sheep with different degrees of ruminal pH. Ruminal lactic acidosis was induced in nine adult crossbred Santa Ines sheep by the administration of 15 g of sucrose per kg/BW. Samples of rumen fluid were collected at the baseline, before the induction of acidosis (*T*_0_) and at six, 12, 18, 24, 48, and 72 hours after the induction for pH measurement using a bench pH meter. During this 72-hour period, all animals had electrodes for the continuous measurement of pH. The results were compared using the Bland-Altman analysis of agreement, Pearson coefficients of correlation and determination, and paired analysis of variance with Student's *t*-test. The measurement methods presented a strong correlation (*r* = 0.94, *P* < 0.05) but the rumen pH that was measured continuously using a telemetry system resulted in lower values than the bench pH meter (overall mean of 5.38 and 5.48, resp., *P* = 0.0001). The telemetry system was able to detect smaller changes in rumen fluid pH and was more accurate in diagnosing both subacute ruminal lactic acidosis and acute ruminal lactic acidosis in sheep.

## 1. Introduction

Rumen lactic acidosis is a nutritional metabolic disease that causes great economic losses in ruminants and can evolve into subacute, acute, or chronic forms. Subacute ruminal lactic acidosis (SARA) is caused by the sudden intake of a high-carbohydrate diet when the rumen has not yet adapted. The intake of this type of food stimulates the rumen microflora to ferment, producing short-chain fatty acids (SCFAs) and lactic acid, which are rapidly used by fermenting bacteria [1]. Acute rumen lactic acidosis (ARLA) is characterised by the excessive intake of soluble carbohydrates and elevated lactic acid concentrations, with a subsequent reduction in ruminal pH and marked clinical manifestations [2, 3].

The rumen is the main organ that is involved in ARLA and SARA, and therefore, this organ is the first to be evaluated in suspected cases of these diseases. The rumen has a heterogeneous environment in terms of the pH because its physiological value presents variation and is influenced by the type of food that is consumed, by water intake and by rumination [1]. The stability of the pH in the rumen is maintained by the relation between the bacterial population, growth substrates that are available in the organ, fermentation products, and the buffering effect of the saliva [3–5].

Among the collection methods of rumen contents for the measurement of ruminal pH, the most common are a ruminal probe, rumenocentesis, and ruminal fistula, which is typically measured using a bench pH meter. Recently, internal continuous sensors that are wired or that are wireless have been used. The continuous measurement system of ruminal pH through these internal sensors has been employed in cannulated cattle to study ruminal metabolism [6–9]. This approach offers a means to study the interaction between different ruminal variables, which enables the mobility of the animal. Moreover, the continuous measurement of ruminal pH can detect rapid fluctuations in variables that are often more difficult to acquire with punctual evaluation [10].

The aim of this study was to compare the measurement of ruminal fluid pH using a continuous telemetry system and a bench pH meter under ARLA, SARA, and normal conditions.

## 2. Materials and Methods

Nine crossbred Santa Ines sheep with an average weight of 45 ± 1.8 kg and age of 24 months were used. All sheep underwent surgery for the implantation of a silicone rumen cannula and were dewormed (Cydectin, Zoetis Animal Health, Campinas, Brazil). After the surgery, the animals passed through a period of 60 days for recovery and adaptation to the new conditions of management and diet.

During the adaptation period and throughout the course of the study, the animals were fed a basal diet that was calculated at 2.7% of body weight; with the dry matter composed of 75% hay coast cross and 25% commercial concentrate pellets (Fri-Sheep 22/70, Nutreco Animal Nutrition, Pitangueiras, Brazil), which was offered twice daily. Sheep had free access to water.

The animals were kept in individual metabolic cages at the Centre for Research in Ruminant Nutritional and Metabolic Diseases, Department of Veterinary Medicine, Faculty of Veterinary Medicine and Animal Science, University of São Paulo (USP). This study was approved by the local Animal Ethics Commission.

The experimental induction of rumen lactic acidosis was performed in all animals through the administration of 15 g of sucrose per kg/BW directly into the rumen according to the protocol described by Kezar and Church [11] and Afonso et al. [12].

In all animals, a continuous telemetry measurement system, which measures the rumen fluid pH every 5 minutes and has a sensitivity of 0.01 pH units, was installed. The system was composed of a submersible electrode (PHE-6510, weighing 120 grams) (Omega Engineering Inc., Connecticut, USA), that was coupled to a data logger (OM-CP-PH10) (Omega Engineering Inc., Connecticut, USA), a transfer cable (OM-CP-IFC110) (Omega Engineering Inc., Connecticut, USA), and a specific software (Omega 2.04.6) (Omega Engineering Inc., Connecticut, USA). The electrode was implanted in the ventral sac of the rumen as described previously by AlZahal et al. [6].

The electrodes and data logger were calibrated using the aforementioned software and standard solutions (Merck, São Paulo, Brazil) with pH values of 4.0 and 7.0, with the calibration procedure performed using the software as indicated by the manufacturer. The system for the continuous measurement electrode was placed in each animal from the baseline and remained in place for 72 hours.

To compare the results that were obtained by the telemetry system, punctual samples of ruminal contents were collected from the ventral sac of the rumen, at the same location of the continuous electrode, at the following times: *T*_0_ (baseline), *T*_6h_ (six hours), *T*_12h_ (twelve hours), *T*_18h_ (eighteen hours), *T*_24h_ (twenty-four hours), *T*_36h_ (thirty-six hours), *T*_48h_ (forty-eight hours), and *T*_72h_ (seventy-two hours) after the sucrose administration. The samples, which contained approximately 100 mL of rumen contents, were collected using a plastic probe that was attached to a vacuum pump and were promptly analysed using a bench pH meter (Model PG 1800, Genhaka, São Paulo, Brazil) with a sensitivity of pH 0.01.

To study the relation between two measurements systems, a Bland-Altman analysis of agreement between methods was performed and the Pearson coefficients of correlation (*r*) and determination (*r* ^2^) were calculated. We also run a paired analysis of variance (ANOVA) with means compared using Student's* t*-test. This ANOVA was performed first globally and then considering only normal (pH ≥ 5.6), SARA (5.6 < pH ≤ 5.0), or ARLA (pH < 5.0) pH ranges. The significance level adopted was 5%. Considering the telemetry system as the gold standard, the sensitivity, and specificity of the conventional method were evaluated separately in SARA and ARLA pH ranges.

## 3. Results and Discussion

**Figure 1:**
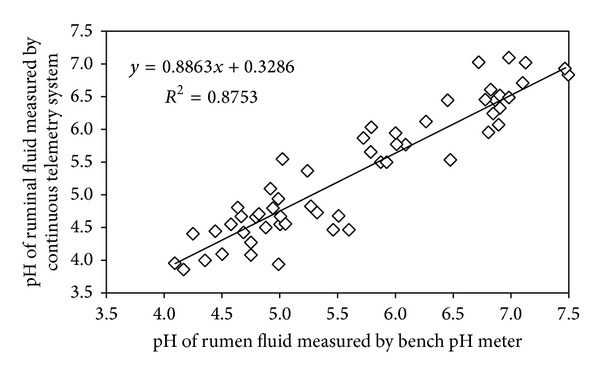
Linear correlation between the rumen fluid pH measurements by the continuous telemetry system and the conventional bench pH meter.

The mean pH values of the rumen fluid were 5.48 and 5.38 for the conventional system and telemetry system, respectively, with significant differences (*P* = 0.0001) between the two methods. Bland-Altman analysis shows a positive bias for the ruminal fluid pH measured thought bench pH meter, but with a strong correlation (*r* = 0.94, *P* < 0.05) between the two methods of measurement, as shown in Figure 1.

Table 1 presents the comparison between the results of the rumen pH between the two measurement methods that were used, considering the overall average, and the results were separated in three different ranges, for the normal pH (pH ≥ 5.6), for acidic pH in cases of SARA (5.6 < pH ≥ 5.0) and for a pH range that was indicative of ARLA (pH < 5.0). The linear correlation obtained using the general pH data follows the equation *y* = 0.8863*x* + 0.3286 (*r* ^2^ = 0.8753) and has a Pearson correlation coefficient of 0.94 (*P* < 0.05). The Pearson correlation coefficients for normal, SARA, and ARLA pH ranges are also presented in Table 1.

Continuous pH measurement systems were evaluated in cattle with high carbohydrate diets to study the changes in rumen pH [6, 7, 9]; however, the pH values observed in those studies were high (greater than 6.0) and were not characteristic of SARA's cases or ARLA. We evaluated the continuous method in more challenging situations, with higher pH fluctuations. In addition, the most studies regarding the continuous measurement of rumen pH have been performed in cattle. In sheep, Kaur et al. [13] evaluated a wireless probe with poor results, and Penner et al. [14] had satisfactory correlation coefficients using a small ruminant ruminal pH measurement system; however, both studies only evaluated a normal pH range.

Regarding the compared analysis of the two methods and using the telemetry system as gold standard, for SARA pH range, the bench pH meter had sensitivity of 1.0 (0.8843 to 1.000, 95% confidence interval) and specificity of 0.8667 (0.6928 to 0.9624, 95% CI), with four false negative results (sheep that had a rumen pH lower than 5.6 using the telemetry system but that had a pH above this threshold using the bench pH meter).

For the ARLA pH range, the sensitivity was 0.9677 (0.8330 to 0.9992 CI) and the specificity was 0.6897 (0.4917 to 0.8472), with nine false negative results (sheep that had rumen pH lower than 5.0 using the telemetry system but that had a pH above this threshold using the bench pH meter) and one false positive (sheep that had rumen pH higher than 5.0 using the telemetry system but that had a pH below this threshold using the bench pH meter). Thus, field cases of SARA and ARLA can be misdiagnosed when conventional methods for rumen fluid pH measures were used.

Similar to previous research, the overall results of pH measurements in different pH ranges were always higher using the bench pH meter compared with the telemetry system measurements [8, 10]. The explanation for this difference is that the telemetry probe remains deep in the ventral sac of the rumen but the ruminal contents obtained for measurement using the bench pH meter, although careful collected, were always contaminated with rumen fluid from more dorsal areas of rumen, and the fluid in this area has proven to have a more alkaline pH [15]. Another factor that may have influenced the pH was that an inevitable loss of CO_2_ and SCFAs occurs during sample collection and measurement using a bench pH meter, which results in a slightly more alkaline pH [15].

Sato et al. [9] and AlZahal et al. [6] found differences in the mean rumen pH between methods of 0.14 and 0.7, respectively. Globally, our mean difference was 0.1; however, when measurements are separately compared, that is, normal, SARA and ARLA pH ranges, the differences were higher (0.22, 0.60 and 0.21, resp.), with a lower degree of significance (*P* < 0.047) for e SARA range when compared with the other pH ranges.

Most likely, this higher difference at the SARA pH range was due to the higher concentration of SCFAs in the fluid when compared with normal and ARLA ranges. Despite the extreme caution during the bench measurement, some SCFAs would volatilise, which would lead to a slight alkalinisation of the sample. In ARLA, lactic acid is predominant, and because lactic acid is not volatile, the sample for the bench pH meter measurement is more stable. These results should be considered for SARA diagnosis by rumen pH measurement because the real value can be smaller than the measured value. In conclusion, the rumen pH measured continuously using a data acquisition telemetry system has measured significantly lower values than those values determined using a bench pH meter under normal conditions and in cases of SARA and ARLA pH ranges, which demonstrates that this method is able to detect minor variations in the rumen pH. The telemetry system was more accurate in diagnosing both SARA and ARLA in sheep.

## Figures and Tables

**Table 1:** Means, standard deviations (SD), and linear correlations of rumen pH as measured by a bench pH meter and by a continuous telemetry system at different pH ranges.

Method	Average pH measured		Normal pH range (>5.6)		SARA pH range (5.6 < pH ≤ 5.0)		ARLA pH range (≤5.0)	
	Mean	SD	Mean	SD	Mean	SD	Mean	SD
pH bench	5.48^A^	0.99	6.64^A^	0.40	5.40^A^	0.14	4.54^A^	0.27
Telemetry system	5.38^B^	1.01	6.42^B^	0.37	4.80^B^	0.44	4.33^B^	0.30

P*	0.001		0.002		0.047		0.001	
*r* ^2^^†^	0.87		0.63		0.53		0.53	
*r* ^‡^	0.94		0.79		0.73		0.73	

Superscripts indicate difference (*P < 0.05) as determined by Student's *t*-test; ^†^determination coefficient; ^‡^correlation coefficient.
